# Clinical impact of ^11^C-Pittsburgh compound-B positron emission tomography in addition to magnetic resonance imaging and single-photon emission computed tomography on diagnosis of mild cognitive impairment to Alzheimer's disease

**DOI:** 10.1097/MD.0000000000023969

**Published:** 2021-01-22

**Authors:** Kazuhiro Kitajima, Kazuo Abe, Masanaka Takeda, Hiroo Yoshikawa, Mana Ohigashi, Keiko Osugi, Hidenori Koyama, Koichiro Yamakado

**Affiliations:** a Department of Radiology; b Department of Neurology, Hyogo College of Medicine; c Department of Internal Medicine, Division of Diabetes, Endocrinology and Metabolism, Hyogo, Japan.

**Keywords:** [^11^C] Pittsburgh compound-B positron emission tomography (^11^C-PiB), Alzheimer's disease (AD), mild cognitive impairment (MCI)

## Abstract

This study aimed to evaluated the clinical impact of adding [^11^C] Pittsburgh compound-B (^11^C-PiB) PET for clinical diagnosis of mild cognitive impairment (MCI) to Alzheimer's disease (AD) dementia.

Twenty six (mean age 78.5 ± 5.18 years, 21 females) AD (n = 7), amnestic MCI (n = 12), non-amnestic MCI (n = 3), vascular dementia, progressive supranuclear palsy (PSP) with frontotemporal dementia (FTD), FTD (n = 1 each), and normal (n = 1) patients underwent ^11^C-PiB-PET, MRI, and SPECT scanning. ^11^C-PiB-PET was compared with MRI and SPECT for clinical impact.

^11^C-PiB-PET showed positivity in 6, 9, and 0 of the AD, amnestic MCI, and non-amnestic MCI patients, respectively, and 0 of those with another disease. Parahippocampal atrophy at VSASD was observed in 5 AD patients, 6 amnestic and PiB-positive MCI patients, 1 amnestic and PiB-negative MCI patient, and 1 vascular dementia patient. Parietal lobe hypoperfusion in SPECT findings was observed in 6, 4, and 2 of those, respectively, as well as 1 each of non-amnestic MCI, vascular dementia, and normal cases. Sensitivity/specificity/accuracy for selecting PiB-positive patients among the 15 MCI patients for ^11^C-PiB-PET were 100% (9/9)/100% (6/6)/100% (15/15), for VSRAD were 66.7% (6/9)/83.3% (5/6)/73.3% (11/15), and for SPECT were 44.4% (4/9)/50.0% (3/6)/46.7% (7/15), while those were 88.9% (8/9)/33.3% (2/6)/66.7% (10/15)/for combined VSRAD and SPECT. ^11^C-PiB-PET accuracy was significantly higher than that of SPECT.

^11^PiB-PET alone may be useful for selecting patients who will progress from MCI to AD in the future, although follow-up study is necessary to clarify the outcome of MCI patients.

## 1 Introduction

Alzheimer's disease (AD) is the most common neurodegenerative disorder in elderly individuals as well as the most common cause of dementia, with steadily increasing numbers. Major pathological features of the disease include the presence of senile plaques in the brain caused by amyloid β-peptide (Aβ) deposition and neurofibrillary tangles containing tau protein. According to the amyloid hypothesis, accumulation of Aβ in the brain is the primary abnormality related to AD pathogenesis, though that reaches a plateau at the time of appearance of clinical symptoms.^[1]^ [^11^C]Pittsburgh compound-B (^11^C-PiB), known as a representative tracer for amyloid-imaging positron emission tomography (PET), binds to Aβ protein, allowing direct visualization of Aβ plaques in vivo.^[2]^ Strong associations of in vivo ^11^C-PiB retention with the results of region-matched quantitative analyses of Aβ plaques have been reported in postmortem studies.^[3]^

Mild cognitive impairment (MCI) is a condition in which the activities of daily life are preserved but memory impairment is beyond the range of normal age-related decline. MCI can be broadly divided into 2 types;

1. amnestic MCI, in which memory decline is the defining feature, and
2. non-amnestic MCI, in which there are predominant deficits in attention, executive function, visuospatial skills, and/or language.^[4]^

MCI is a transitional phase between a normal state and dementia caused by a heterogeneous group of disorders, and in many cases is progressive and represents a predementia stage of AD.^[5–7]^ For treatment strategies aimed at slowing disease progression, early detection of AD is of utmost importance. However, neither pathology type nor individual prognosis can be reliably established on the basis of clinical symptoms alone. Some studies have found a correlation between clinical course and PiB retention in MCI patients, and those patients who converted to AD were shown to have higher PiB retention as compared to non-conversion cases.^[8–11]^

In Japan, magnetic resonance imaging (MRI) and single photon emission computed tomography (SPECT) are the primary imaging modalities used for diagnosis of dementia in daily clinical practice. In the present study, we evaluated the impact of addition of ^11^C-PiB PET to those routinely employed imaging methods for clinical diagnosis of MCI and AD.

## 2 Materials and methods

### 2.1 Patients

All subjects who participated in the present study were recruited from patients who visited the outpatient clinic of the Department of Neurology, Hyogo College of Medicine Hospital, between January 2018 and March 2020. The cohort totaled 26 (21 females, 5 males; mean age 78.5 ± 5.18 years), including 7 with AD, 12 with amnestic MCI, 3 with non-amnestic MCI, 1 with vascular dementia, 1 with progressive supranuclear palsy (PSP) with frontotemporal dementia (FTD), and 1 with FTD, as well as 1 diagnosed as normal. This clinical PET study was conducted after receiving approval from the ethics committee of the Hyogo College of Medicine. Written informed consent for participation public release of data was obtained from all enrolled patients. Prior to imaging examinations (MRI, SPECT, ^11^C-PiB PET), a comprehensive diagnosis of dementia was performed by Japanese-board certified physicians using clinical diagnostic guidelines of the Japanese Society of Neurology as well as others. A diagnosis of MCI was based on the criteria of the MCI key symposium in Stockholm.^[12]^ Eligibility criteria for AD diagnosis used in the present study were based on those presented by the National Institute of Neurological and Communicative Disorders and Stroke (NINCDS) and the Alzheimer's Disease and Related Disorders Association (ADRDA).^[13]^ The interpretation of scan finding was performed in a “blind” manner for each imaging modality.

### 2.2 Neuropsychological assessment

For assessment of cognitive function, the Mini-Mental State Examination (MMSE),^[14]^ Frontal Assessment Battery (FAB),^[15]^ and Montreal Cognitive Assessment (MoCA-J)^[16]^ were performed by qualified clinical psychologists.

### 2.3 MRI

All MRI examinations including whole-brain volumetric imaging with 3-D gradient refocused echo sequence (magnetization-prepared rapid gradient echo) were performed using 2 different 3.0 Tesla imaging systems; a Magnetom Skyra (Siemens, Erlangen, Germany) (TR = 1800 ms, TE = 2.92 ms, flip angle = 10°, voxel size = 0.9 × 0.9 × 1.0 mm^3^, field of view = 240 mm, matrix = 256 × 256, slice thickness = 1.0 mm with no gap) and an Achieva (Philips Healthcare, Best, The Netherlands) (TR = 7.4 ms, TE = 3.5 ms, flip angle = 10°, voxel size = 1.0 × 1.0 × 1.0 mm^3^, field of view = 256 mm, matrix = 256 × 256, slice thickness = 1.0 mm with no gap).

MR images were visually interpreted by an experienced neuroradiologist. In addition, to support visual assessments for evaluating medial temporal atrophy, 3D-T1WI images were analyzed with the widely used software package Voxel-based specific Regional Analysis System for Alzheimer's Disease (VSRAD advance: Eisai Co, Ltd, Tokyo, Japan).^[17]^ VSRAD automatically calculates Zscore [(normal control average of voxel level - patient voxel level)/normal control standard deviation (SD)], hippocampal atrophy on VSRAD (HA-V) [(number of voxels judged to have Z-score >2/number of all voxels in volume of hippocampus) × 100%], and whole-brain atrophy [(number of voxels judged to have Z-score >2/number of all voxels in volume of whole brain) × 100%]. “Voxel level” means gray matter density per unit voxel and 1 voxel means 2 mm × 2 mm × 2 mm. Atrophy of the hippocampus, shown by a Z-score >2 as compared with the whole brain, was considered to be a positive finding.

### 2.4 ^123^I-IMP SPECT

All patients underwent SPECT with N-isopropyl-*p*-[^123^I]-iodoamphetamine ([^123^I]-IMP) (Mediphysics Pharma, Tokyo, Japan), injected at a maximum dose of 222 MBq (6 mCi). The SPECT/CT scanner (NM/CT670; GE Healthcare, Waukesha, WI, USA) was equipped with a low-energy high-resolution collimator and the following settings were used to acquire SPECT data: 30 projections per head over 180° on a 512 × 512 matrix, acquisition time of approximately 30 minutes. For attenuation correction, we used CT images obtained with SPECT/CT.

Uptake patterns in ^123^I-IMP SPECT images were visually interpreted by a board-certified nuclear medicine physician for detecting neuronal injury. The AD pattern was diagnosed by visual assessments to identify any decrease in regional cerebral blood flow (rCBF) in the parietotemporal regions, posterior cingulate gyrus, and/or precuneus. To support visual assessment findings and for objective evaluation of rCBF distribution, mean Z-scores (normal mean-individual value/normal SD) were calculated using a 3-D stereotactic surface projection (3D-SSP) technique^[18]^ to generate z-score maps, with the software package 3D-SSP Z-Graph (Aze Virtalplace, Kanagawa, Japan) utilized for statistical analyses of all SPECT images. A decrease in rCBF >1.64 SDs based on Z-score was considered to be a positive finding.

### 2.5 ^11^C-PiB PET

^11^C-PiB was injected intravenously at a dose of 555 ± 125 MBq/kg at 50 minutes before the start of brain PET scanning. All imaging examinations were performed with a combined PET/CT scanner (Discovery iQ HD; GE Healthcare, Waukesha, WI, USA). ^11^C-PiB attenuation-corrected PET images were reconstructed using CT data and Q.clear reconstruction algorithm whether the data sets were corrected for high counting losses given that applied ^11^C-PiB Doses were about 555MBq, resulting in images with a 192 × 192 × 79 matrix and 2.6 × 2.6 × 3.2-mm voxels, with no Gaussian filter smoothing.

^11^C-PiB images were visually interpreted by a board-certified nuclear medicine physician. ^11^C-PiB deposition was categorized as ^11^C-PiB uptake-positive (higher PiB retention in cortex than that in white matter) or ^11^C-PiB uptake-negative (lower PiB retention in cortical areas), according to standard visual assessment criteria.^[19]^ For visual interpretation, the raters evaluated regional ^11^C-PiB uptake for each of 4 cortical areas on each side (frontal, lateral temporal, and lateral parietal lobes, precuneus/posterior cingulate gyrus) as positive or negative regional uptake, and evaluated whether or not accumulation was higher in the cerebral cortex than in white matter. A positive amyloid scan was defined when there was at least 1 positive area in any of the above 4 cortical regions. To support visual assessment findings and for objective evaluation of evidence of abnormal ^11^C-PiB accumulation, the mean cortical standardized uptake value ratio (SUVR) to the cerebellar cortex was used. Utilizing the CortexID Suite (GE Healthcare, Waukesha, WI, USA) software package, the region of interest template included the frontal cortex, lateral temporal cortex, parietal cortex, posterior cingulate, precuneus, occipital cortex, and striatum as the target regions, and the cerebellar cortex as the reference region. Decision of positive or negative visual interpretation was dichotomized by an SUVR cut-off value of 1.5.^[20]^

### 2.6 Statistical analysis

The diagnostic performance of ^11^C-PiB PET was compared with that of VSRAD, SPECT, and combined VSRAD and SPECT using Mc Nemar test. Differences for age, MMSE score, FAB score, and MoCA-J score between ^11^C-PiB-positive and -negative MCI patient groups and between AD and MCI patients were compared using Student *t* test, with *P* < .05 considered to indicate statistical significance.

## 3 Results

**Figure 1:**
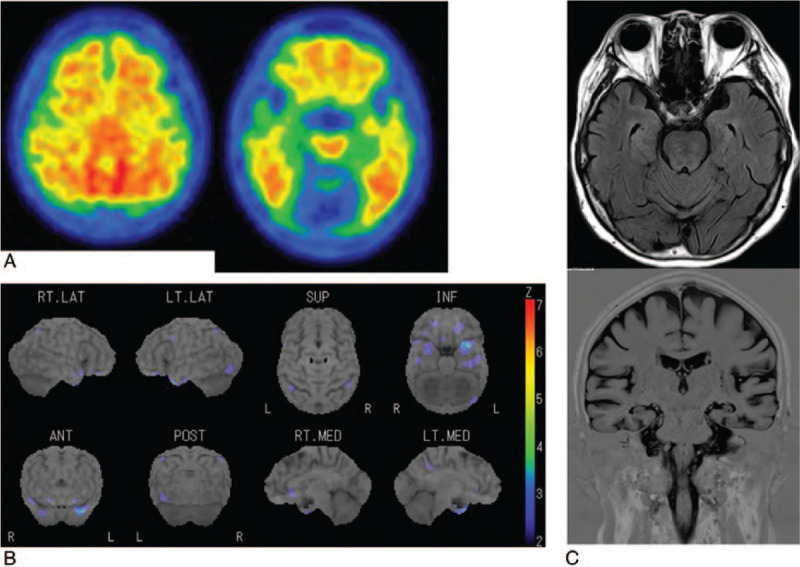
A 78-year-old woman with amnestic MCI (Case 16). a. ^11^C-PiB PET shows positive amyloid burden with, a mean cortical standardized uptake value ratio (SUVR) of 1.90. b. ^123^I-IMP SPECT and color-coding representing the statistical significance of decrease in regional cerebral blood flow as compared to normal does not show hypoperfusion in any parietotemporal regions, posterior cingulate gyrus and precuneus. c. Axial and coronal MRI shows no hippocampal atrophy, with a Z-score of 1.88.

**Figure 2:**
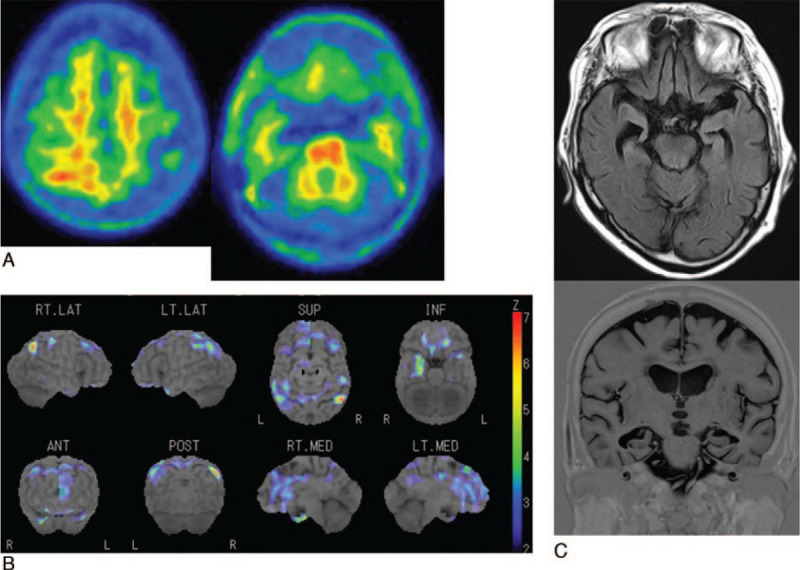
A 81-year-old woman with vascular dementia (Case 23). a. ^11^C-PiB PET shows negative amyloid burden with, a mean cortical standardized uptake value ratio (SUVR) of 1.40. b. ^123^I-IMP SPECT and color-coding representing the statistical significance of decrease in regional cerebral blood flow as compared to normal shows hypoperfusion in both the parietotemporal regions, posterior cingulate gyrus and precuneus. c. Axial and coronal MRI shows severe atrophy of both hippocampus, with a Z-score of 3.48.

Patient characteristics are shown in Table 1. ^11^C-PiB PET positivity was found in 6 (85.7%) of 7 AD patients, 9 (60.0%) of 15 MCI patients, and 0 (0%) of 4 patients with another disease. Hippocampal atrophy shown by VSRAD was observed in 5 (71.4%), 7 (46.7%), and 1 (25.0%) of those groups, while SPECT results showed hypoperfusion of the parietal lobe in 6 (85.7%), 7 (46.7%), and 2 (50.0%), respectively. Results obtained with a combination of VSRAD and SPECT (VSRAD or SPECT showing an AD pattern defined as positive) showed abnormalities in 6 (85.7%), 12 (75.0%), and 2 (50.0%), respectively. Findings of 2 representative cases are presented in Figures 1 and 2.

**Table 1:** Patient characteristics.

								MRI			
Case	Age	Sex	MMSE	FAB	MoCA-J	Clinical diagnosis	PiB-PET	SUVR	(VSRAD)	Z-Score	SPECT
1	79	F	19	5	16	AD	Positive	2.55	High	3.0	High
2	83	F	17	3	1	AD	Positive	1.93	High	3.08	High
3	86	M	27	16	22	AD	Positive	1.92	High	2.5	High
4	83	F	16	15	14	AD	Positive	2.31	High	2.95	High
5	71	F	18	11	16	AD	Positive	2.67	WNL	0.88	High
6	73	F	19	16	12	AD	Positive	1.80	WNL	1.58	WNL
7	82	M	23	14	19	AD or AGD	Negative	1.29	High	2.83	High
8	74	F	29	16	28	MCI (amnestic)	Positive	1.66	High	3.18	High
9	83	F	24	16	18	MCI (amnestic)	Positive	2.37	High	2.51	High
10	85	F	22	17	21	MCI (amnestic)	Positive	2.62	High	2.08	WNL
11	72	F	23	15	8	MCI (amnestic)	Positive	1.82	High	2.78	WNL
12	75	F	21	15	22	MCI (amnestic)	Positive	2.17	High	3.1	WNL
13	85	M	26	17	18	MCI (amnestic)	Positive	1.59	High	2.61	WNL
14	78	F	25	18	21	MCI (amnestic)	Positive	1.96	WNL	1.58	High
15	78	F	25	17	19	MCI (amnestic)	Positive	1.66	WNL	1.05	High
16	78	F	27	17	21	MCI (amnestic)	Positive	1.90	WNL	1.88	WNL
17	83	F	23	18	23	MCI (amnestic)	Negative	1.26	High	3.58	WNL
18	82	F	22	14	18	MCI (amnestic)	Negative	1.12	WNL	1.79	High
19	80	F	25	15	22	MCI (amnestic)	Negative	1.48	WNL	1.84	High
20	82	F	25	12	3	MCI (nonamnestic)	Negative	1.16	WNL	1.01	High
21	68	F	29	13	25	MCI (nonamnestic)	Negative	1.14	WNL	0.33	WNL
22	79	F	26	13	20	MCI (nonamnestic)	Negative	1.06	WNL	1.02	WNL
23	81	F	21	11	18	Vascular dementia	Negative	1.40	High	3.48	High
24	74	M	13	5	11	PSP with FTD	Negative	1.48	WNL	1.08	WNL
25	78	F	17	8	18	FTD	Negative	1.05	WNL	1.78	WNL
26	68	M	26	18	25	Normal	Negative	1.31	WNL	1.02	High

Of the 15 MCI patients, ^11^C-PiB PET showed positivity in 9 (75.0%) of 12 with amnestic MCI, whereas all 3 non-amnestic MCI patients were negative. VSRAD showed hippocampal atrophy in 6 (66.7%) of the 9 amnestic MCI patients with positive ^11^C-PiB PET findings and 1 (33.3%) in 3 of those with negative ^11^C-PiB PET findings, and that was seen in 0 (0%) of 3 non-amnestic MCI patients, while in those same groups SPECT showed an AD pattern in 4 (44.4%), 2 (66.7%), and 1 (33.3%), respectively. As for the combined VSRAD and SPECT method, an AD pattern was shown in 8 (88.9%), 3 (100%), and 1 (33.3%), respectively

Table 2 presents results showing sensitivity, specificity, and accuracy to indicate diagnostic performance for differentiating AD and PiB-positive MCI (n = 16) from PiB-negative MCI and other diseases (n = 10). Those were 93.8% (15/16), 100% (10/10), and 96.2% (25/26) for ^11^C-PiB PET, 68.8% (11/16), 80.0% (8/10), and 73.1% (19/26) for VSRAD, 62.5% (10/16), 50.0% (5/10), and 57.7% (15/26) for SPECT, and 87.5% (14/16), 40.0% (4/10), and 69.3% (18/26) for combined VSRAD and SPECT, respectively. Among the 4 imaging methods examined, ^11^C-PiB PET showed the highest levels of sensitivity and specificity, and accuracy was significantly higher as compared to VSRAD (*P* = .041), SPECT (*P* = .0044), and combined VSRAD and SPECT (*P* = .023). The specificity of SPECT was low, while the combined VSRAD and SPECT method showed a relatively high level of sensitivity and low level of specificity.

**Table 2:** Performance of differentiating AD and PiB-positive MCI (n = 16) from PiB-negative MCI and other disease (n = 10).

	PiB-PET	MRI (VSRAD)	SPECT	MRI+SPECT
Sensitivity	15/16 (93.8%)	11/16 (68.8%)	10/16 (62.5%)	14/16 (87.5%)
Specificity	10/10 (100%)	8/10 (80%)	5/10 (50%)	4/10 (40%)
PPV	15/15 (100%)	11/13 (84.6%)	10/15 (66.7%)	14/20 (70%)
NPV	10/11 (90.9%)	8/13 (61.5%)	5/11 (45.5%)	4/6 (66.7%)
Accuracy	25/26 (96.2%)	19/26 (73.1%)	15/26 (57.7%)	18/26 (69.3%)

Table 3 shows results indicating sensitivity, specificity, and accuracy for selecting the 9 PiB-positive patients among the 15 with MCI. Those values for ^11^C-PiB PET were 100% (9/9), 100% (6/6), and 100% (15/15), for VSRAD were 66.7% (6/9), 83.3% (5/6), and 73.3% (11/15), for SPECT were 44.4% (4/9), 50.0% (3/6), and 46.7% (7/15), and for combined VSRAD and SPECT were 88.9% (8/9), 33.3% (2/6), and 66.7% (10/15), respectively. The accuracy of ^11^C-PiB PET was significantly higher than that of SPECT (*P* = .013), and the combined VSRAD and SPECT method showed a relatively high sensitivity and low specificity.

**Table 3:** Performance of selecting 9 PiB-positive patients in 15 MCI patients.

	PiB-PET	MRI (VSRAD)	SPECT	MRI+SPECT
Sensitivity	9/9 (100%)	6/9 (66.7%)	4/9 (44.4%)	8/9 (88.9%)
Specificity	6/6 (100%)	5/6 (83.3%)	3/6 (50.0%)	2/6 (33.3%)
PPV	9/9 (100%)	6/7 (85.7%)	4/7 (57.1%)	8/12 (66.7%)
NPV	9/9 (100%)	5/8 (62.5%)	3/8 (37.5%)	2/3 (66.7%)
Accuracy	15/15 (100%)	11/15 (73.3%)	7/15 (46.7%)	10/15 (66.7%)

Of the 15 MCI patients, the mean age of those with PiB-positive (n = 9) and PiB-negative (n = 6) findings was 78.7 ± 4.7 and 79.0 ± 5.6 years, respectively, which was not a significant difference (*P* = .45). In those groups, the mean MMSE score was 24.7 ± 2.5 and 25.0 ± 6.0 (*P* = .40), mean FAB score was 16.4 ± 1.0 and 14.2 ± 2.1 (*P* = .99), and mean MoCA-J score was 19.6 ± 5.3 vs 18.5 ± 8.0 (*P* = .62), respectively, with no significant differences noted.

In the comparison between AD (n = 7) and MCI (n = 15) patients, the mean age of those with groups was 79.6 ± 5.6 and 78.8 ± 4.9 years (*P* = .75), and mean MoCA-J score was 14.3 ± 6.7 vs 19.1 ± 6.2 (*P* = .11), respectively, with no significant differences. On the other hand, in those groups, the mean MMSE score was 19.9 ± 3.8 and 24.8 ± 2.4 (*P* = .014), and mean FAB score was 11.4 ± 5.4 and 15.5 ± 1.9 (*P* = .014), respectively, with significant differences.

## 4 Discussion

In the present study, 9 of 15 (60%) MCI patients were found to be positive for PiB. In previous reports, approximately 52% to 68% of examined MCI patients were shown to have amyloid deposition.^[8–11,21]^ In one of those, a study that analyzed ^11^C-PiB PET findings, it was noted that 82% of MCI patients with amyloid deposition demonstrated clinical conversion to AD dementia over a 3-year follow-up period as compared to only 7% of MCI patients without an amyloid burden.^[8]^ Using amyloid PET imaging, it may be possible predict the possibility of MCI converting to AD dementia, allowing for early appropriate treatment to be given. Kikukawa et al [11] explored ^11^C-PiB PET and brain SPECT for the development of dementia in 28 patients with amnestic MCI at an 18-month follow-up study and demonstrated that the rate of conversion to dementia was significantly higher in the PiB-positive/equivocal group (74%) than in the PiB-negative group (33%) (*P* = .041). They also wrote MCI patients with an AD-characteristic pattern of reduced CBF had a higher PiB-positive/equivocal rate (82%) than those with a non-AD pattern (20%) (*P* = .018), and patients with an AD pattern had a higher conversion rate (82%) than those with a non-AD pattern (40%) (*P* = .094) without significant difference. SPECT imaging has a low spatial resolution and the limitation of cold spot imaging as compared to ^11^C-PiB PET imaging.

Moreover, amnestic MCI has been reported to have a higher conversion rate to dementia, especially AD, as compared to non-amnestic MCI patients.^[4,6,7]^ It is interesting to note that in our series the rate of amyloid deposition shown by ^11^C-PiB PET amnestic MCI patients was 75% (9/12) as compared to 0% (0/3) for the non-amnestic MCI cases. Analysis of follow-up findings regarding the clinical outcome of amnestic and non-amnestic MCI patients will be necessary to clarify whether ^11^C-PiB PET results can serve as a surrogate marker.

Omachi et al [21] compared ^11^C-PiB PET, MRI, and brain SPECT in 25 patients with MCI in similar to our series and demonstrated that 18 were MRI and SPECT-positive, 13 were ^11^C-PiB-positive, and 10 who were both MRI and SPECT- and ^11^C-PiB-positive were categorized as having “MCI due to AD-high likelihood”. Yamane et al [20] evaluated inter-rater variability of visual interpretation and comparison with quantitative evaluation of ^11^C-PiB PET amyloid images in a total 162 scans including 45 mild AD, 60 MCI, and 57normal congnitive control cases and demonstrated that inter-rater agreement was almost perfect in ^11^CPiB PET scans (Cohen κ= 0.88–0.89) and positive or negative decision by visual interpretation was dichotomized by a cut-off value of mean cortical SUVR to the cerebellar cortex = 1.5. They also stated that as some cases of disagreement among raters tended to show low mean cortical SUVR, referring to quantitative method may facilitate correct diagnosis when evaluating images of low amyloid deposition.

We found that MRI, SPECT, and combined MRI and SPECT were satisfactory for differentiation of AD and PiB-positive MCI cases from others, as well as selection of PiB-positive patients among the MCI cases as compared with ^11^C-PiB PET. On the other hand, we were not able to differentiate PiB-positive from PiB-negative MCI patients using neuropsychological assessments (MMSE, FAB, MoCA-J scores), similar to a previous report.^[11]^ As a result, we consider that ^11^C-PiB PET may be clinically useful to select MCI patients who will progress to AD in the future, whereas results obtained with MRI, SPECT, or those neuropsychological assessments are not adequate.

One AD patient (case 7) with negative PiB-PET results was clinically suspected to have argyrophilic grain disease (AGD). PiB-negative cases likely included those with other tauopathies related to late-onset dementia, such as neurofibrillary tangle-predominant dementia (NFTD).^[22,23]^ On the other hand, it has been reported that neocortical diffuse amyloid plaque foci were found at autopsy in at least some patients with negative amyloid PET imaging results,^[24,25]^ which suggests that amyloid PET imaging may not be able to detect amyloid deposition in all cases. ^11^C-PiB binds to insoluble (fibrillar) Aβ in compact or core plaques and cerebral amyloid angiopathy in a highly selective manner, though may fail to bind to soluble (non-fibrillar) Aβ in diffuse plaques and Aβ oligomers.^[26,27]^ Thus, additional pathological evaluations are necessary for PiB-negative patients for determination of diagnosis.

This study has several limitations, including its single-institution design and small number of subjects. Furthermore, no follow-up information regarding the clinical outcome of the MCI patients was available to clarify whether ^11^C-PiB PET results can serve as a surrogate marker. Additionally, in Japan, ^18^F-fluorodeoxyglucose (^18^F-FDG) PET is not covered by the national health insurance program for detection of dementia, thus the more widely available MRI and SPECT are the primary modalities used for such cases.^[21]^ The diagnostic capability of ^18^F-FDG PET and SPECT for AD was recently reported to be similar.^[27]^ Perfusion SPECT evaluation is not included in the NIA-AA research criteria for AD dementia, though can be used as substitute for ^18^F-FDG PET. To evaluate the impact of ^11^C-PiB PET on routine clinical diagnosis of dementia and MCI in Japan, SPECT rather than ^18^F-FDG PET should be used.

In conclusion, amyloid PET imaging results may be useful to predict the possibility of MCI converting to AD dementia, allowing physicians to consider starting treatment early. A follow-up study will be needed to clarify the outcomes of affected MCI patients.

## Author contributions

**Conceptualization:** Kazuhiro Kitajima, Kazuo Abe, Hiroo Yoshikawa, Koichiro Yamakado.

**Data curation:** Kazuhiro Kitajima, Kazuo Abe, Masanaka Takeda, Hiroo Yoshikawa, Mana Ohigashi, Keiko Osugi, Hidenori Koyama.

**Funding acquisition:** Kazuhiro Kitajima.

**Investigation:** Kazuhiro Kitajima.

**Methodology:** Kazuhiro Kitajima, Kazuo Abe, Masanaka Takeda, Hiroo Yoshikawa.

**Project administration:** Kazuo Abe.

**Supervision:** Koichiro Yamakado.

**Writing – original draft:** Kazuhiro Kitajima.

**Writing – review & editing:** Kazuhiro Kitajima, Kazuo Abe, Masanaka Takeda, Hiroo Yoshikawa, Mana Ohigashi, Keiko Osugi, Hidenori Koyama, Koichiro Yamakado.
